# Factors influencing Anti-SARS-CoV-2 IgG levels after vaccination in breast cancer patients

**DOI:** 10.1007/s12672-026-04504-4

**Published:** 2026-02-04

**Authors:** Pei-Wei Shueng, Wen-Chien Ting, Chi-Chang Chang, Wen-Wei Chang, Yi-Ju Tseng, Chin-Fang Chang, Gin-Den Chen

**Affiliations:** 1 Department of Radiation Oncology, Division of Radiation Oncology, Far Eastern Memorial Hospital, New Taipei City, Taiwan; 2 Faculty of Medicine, School of Medicine, National Yang-Ming University, Taipei, Taiwan; 3 Division of Colorectal Surgery, Department of Surgery, Chung Shan Medical University Hospital, Taichung City, 402306 Taiwan; 4 School of Medicine, Chung Shan Medical University, Taichung City, 402306 Taiwan; 5 Department of Medical Informatics, Chung Shan Medical University & IT Office, Chung Shan Medical University Hospital, Taichung, 402306 Taiwan; 6 Department of Information Management, Ming Chuan University, Taoyuan, 111005 Taiwan; 7 Department of Biomedical Sciences, Chung Shan Medical University, Taichung, 402306 Taiwan; 8 Department of Medical Research, Chung Shan Medical University Hospital, Taichung, 402306 Taiwan; 9 Department of Computer Science, National Yang Ming Chiao Tung University, Hsinchu, Taiwan; 10 Computational Health Informatics Program, Boston Children’s Hospital, Boston, MA USA; 11 Rayon Clinic, Taichung, Taiwan; 12 Department of Otorhinolaryngology, Head and Neck Surgery, Taichung Hospital Ministry of Health and Welfare, Taichung, Taiwan; 13 Rong Hsing Research Center For Translational Medicine, National Chung Hsing University, Taichung, Taiwan; 14 Department of Obstetrics and Gynecology, Chung Shan Medical University Hospital, Taichung, Taiwan; 15 Department of Obstetrics and Gynecology, Chung Shan Medical University, Taichung, Taiwan

**Keywords:** COVID-19, SARS-CoV-2, Breast cancer, Seroconversion, Vaccination response

## Abstract

**Background/objectives:**

Breast cancer patients may exhibit impaired vaccine-induced immunity due to disease progression and treatment-related immunosuppression. This study examined clinical factors associated with anti-SARS-CoV-2 IgG responses post-COVID-19 vaccination, with a focus on disease stage and treatment timing.

**Methods:**

We analyzed 63 breast cancer patients vaccinated between 2021 and 2022. Clinical data—including tumor size, stage, lymph node involvement, estrogen receptor (ER) status, and treatments (chemotherapy, radiation)—were collected. Serum anti-SARS-CoV-2 IgG levels were assessed after each vaccine dose.

**Results:**

After the first dose, lower IgG titers were observed in patients receiving chemotherapy, with lymph node involvement, or left-sided tumors. Higher titers were associated with larger tumors (≥ 2 cm), Stage > 2 A, ER positivity, and radiation therapy, possibly due to Th2-skewed responses. Longer diagnosis-to-vaccination intervals enhanced responses. No stage-related differences were found after the second dose, likely due to a ceiling effect. mRNA vaccines tended to elicited higher titers than adenoviral vector vaccines. Reported adverse effects were mild.

**Conclusions:**

Tumor stage, ER status, radiation, and vaccination timing influence humoral responses in breast cancer patients. These findings highlight the need for tailored vaccine strategies, including the timing of mRNA vaccines, to optimize protection in this population.

## Introduction

Since the COVID-19 pandemic began, it has resulted in over 700 million infections and over 7 million deaths globally since 2019 [1]. Overall, vaccination is essential to safeguard vulnerable populations from severe illness and lower the spread of lethal infectious diseases. The COVID-19 vaccines currently available are mRNA, adenovirus vector, and protein subunit. Meanwhile, cancer continues to be a major cause of death globally, responsible for around 9.7 million fatalities each year [2].

Patients with cancer who contract COVID-19 face higher death rates, more frequent admissions to intensive care, and a greater chance of experiencing severe symptoms compared to those without cancer [3]. Studies show that cancer patients have a higher susceptibility to opportunistic infections [4] and could show less effective vaccine-induced immune responses. Research indicates that cancer patients experience lower seroconversion rates following COVID-19 vaccination compared to healthy individuals [5]. Approximately 75% of cancer patients hospitalized with COVID-19 have received the mRNA vaccine, compared to around 90% immunocompetent individuals, before the Omicron variant became dominant [6].

Elements leading to reduced vaccine effectiveness in cancer patients are older age [7], male sex [8], and type of vaccine [9]. In breast cancer patients specifically, those receiving chemotherapy demonstrated the lowest seroconversion rates (81.8%) among all treatment groups [10]. Patients treated with CDK4/6 inhibitors showed reduced neutralizing antibody titers against SARS-CoV-2 variants, though seroconversion remained achievable [11]. Additional breast cancer-specific factors that may influence vaccine response include concurrent corticosteroid administration during chemotherapy, which has been associated with significantly reduced seroconversion rates and lower antibody titers [12]. In contrast, patients receiving endocrine therapy alone demonstrated high seroconversion rates and maintained relatively preserved immune responses to vaccination compared to those receiving chemotherapy [10, 13]. This study intends to explore the factors that influence anti-SARS-CoV-2 antibody levels in breast cancer patients after COVID-19 exposure.

## Materials and methods

### Study design and participants

We gathered and analyzed the vaccination records of breast cancer patients who received their initial and booster SARS-CoV-2 vaccinations between 2021 and 2022, utilizing data from the cancer registry at Chung Shan Medical University Hospital (Taichung City, Taiwan). Additionally, we recruited healthy donors from Jen-Ai Hospital (Taichung City, Taiwan), to serve as controls. The healthy donors were individuals aged over 20 years with no history of cancer or immunosuppressive conditions. This study was approved by the Institutional Review Board (IRB) of Chung Shan Medical University Hospital (approval no. CS2-21150) and the IRB of Jen-Ai Hospital (approval no. 111 − 28). All participants provided written informed consent prior to participation, and all personal identifiers were removed from the data to ensure confidentiality prior to submission for publication.

The inclusion criteria comprised women over 20 years of age with diagnosed invasive breast cancer who had started, completed, or been treated for their condition. Their treatment regimen could include local surgery and radiation therapy or systemic therapies such as chemotherapy, small molecule targeted therapy, immunotherapy, or hormonal therapy. Patient demographics (e.g., age or BMI), treatment history (e.g., receipt of chemotherapy, hormonal therapy, or other systemic treatments), and tumor characteristics (e.g., tumor size, ER/PR/HER2 status, etc.) were extracted from the national cancer registry database, which provided binary information on receptor expression and treatment receipt (yes/no) but not detailed regimens or predefined molecular subtypes. Exclusion criteria encompassed immunosuppressed individuals, such as those with HIV or those whose systemic chemotherapy treatments involved cyclical chemotherapy or immunosuppressive agents. Additional exclusions included patients with an estimated life expectancy of less than 3 months and subjects infected with the SARS-CoV-2 virus (those confirmed by PCR testing or who have recovered). During the enrollment period, participants received one of three available COVID-19 vaccines (AZD1222 from AstraZeneca, BNT162b2 from Pfizer-BioNTech, or mRNA-1273 from Moderna) for both doses, with possible mixed regimens (e.g., different types for first and second doses) due to evolving vaccine availability; these were analyzed as categorical variables for post-second-dose outcomes. Both breast cancer patients and healthy donors agreed to provide whole blood samples during the third week (more than 14 days) after their first COVID-19 vaccine dose and in the second week (more than 7 days) following the second dose. In the second blood draw, participants were also asked to complete a questionnaire regarding any side effects experienced over the prior period. This study was supported by Chung Shan Medical University Hospital (grant number: CSH-2022-D-010).

### Determination of serum Anti-SARS-CoV-2 IgG levels

Whole blood samples from study participants were collected and placed in EDTA tubes. The sera were centrifuged at room temperature at 800 × g for 10 min. Anti-SARS-CoV-2 IgG titer levels in the sera were measured using the AdviseDx SARS-CoV-2 IgG II reagent (Abbott Laboratories, Abbott Park, IL, USA), which has a threshold of 50.0 AU/mL per the manufacturer’s instructions.

### Statistical analysis

All participants with available data were included in the analyses of anti-SARS-CoV-2 antibody titers. Demographic and clinical information was gathered from the cancer registry database. Patient-reported side effects were recorded during follow-up visits. Continuous variables are represented as medians along with interquartile ranges (IQRs). Wilcoxon’s test was used to evaluate differences in anti-SARS-CoV-2 antibody titer levels, and the resulting p-values were adjusted for multiple comparisons using the Benjamini-Hochberg method. Generalized linear modeling utilized the Gaussian distribution method and log-fold link for the multivariate analysis. Calculations were performed in R (version 4.1.3) using the rstatix (version 0.7.0) and gtsummary (version 1.5.2) packages. After applying the Benjamini- Hochberg correction for multiple comparisons, the significance threshold for the resulting p-values was set to less than 0.05.

## Results

We enrolled 63 patients with breast cancer and 43 healthy controls to understand their seroconversion effectiveness following COVID-19 vaccination. A summary of patient demographics and clinical characteristics can be found in Table 1. The types of vaccines administered include an adenoviral vector vaccine (ChAdOx1 nCoV19, noted as AZD1222) and two mRNA vaccines (BNT162b2 and mRNA-1273). Patient sera were collected during the second week after the first dose and the first week after the second dose to evaluate anti-SARS-CoV-2 antibody titers, as determined by the AdviseDx SARS-CoV-2 IgG II reagent protocol. The vaccine doses administered were from different brands based on current availability (Table 1).

**Table 1:** Patient characteristics

Characteristic	*N* (%) / median (IQR)^a^
Patient, n	63
Age (years)	51 (46, 57)
BMI	
< 18.5	3 (4.8%)
≧ 18.5 and < 24	26 (41%)
≧ 24	28 (44%)
Unknown	6 (9.5%)
Laterality	
Right	27 (43%)
Left	36 (57%)
Behavior	
Carcinoma in situ	5 (7.9%)
Invasive carcinoma	58 (92%)
Tumor size	
< 2 cm	18 (29%)
≧ 2 cm	37 (59%)
Unknown	8 (13%)
Regional Lymph nodes positive	
No	38 (60%)
Yes	21 (33%)
Unknown	4 (6.3%)
Pathologic stage	
Stage ≦ 2 A	48 (76%)
Stage > 2 A	6 (9.5%)
Unknown	9 (14%)
Recurrence	
No	59 (94%)
Local and regional	4 (6.3%)
ER	
Negative	10 (16%)
Positive	42 (67%)
Unknown	11 (17%)
PR	
Negative	6 (9.5%)
Positive	46 (73%)
Unknown	11 (17%)
HER2	
Negative	35 (56%)
Positive	18 (29%)
Unknown	10 (16%)
Surgery	61 (97%)
Surgical margins of the primary site	12 (19%)
Radiation therapy and dose to CTV_H^b^	
No radiation therapy	24 (38%)
≦ 4500 cGy	16 (25%)
> 4500 cGy	23 (37%)
Chemotherapy	57 (90%)
Hormone therapy	43 (68%)
Targeted therapy and medication	
No targeted therapy	51 (81%)
Trastuzumab	4 (6.3%)
Trastuzumab + Pertuzumab	7 (11%)
Trastuzumab + Pertuzumab + Neratinib	1 (1.6%)
Time from diagnosis to vaccination (years)	5.00 (3.00, 7.00)
Vaccine brands^c^	
AZD1222 + AZD1222	6 (9.5%)
AZD1222 + BNT162b2	1 (1.6%)
AZD1222 + mRNA-1273	1 (1.6%)
AZD1222 + NA^d^	1 (1.6%)
BNT162b2 + BNT162b2	24 (38%)
BNT162b2 + mRNA-1273	4 (6.3%)
BNT162b2 + AZD1222	1 (1.6%)
BNT162b2 + NA^d^	3 (4.8%)
mRNA-1273 + mRNA-1273	14 (22%)
mRNA-1273 + BNT162b2	4 (6.3%)
mRNA-1273 + NA^d^	4 (6.3%)
IgG after the first dose	610 (258, 1,082)
IgG after the second dose^d^	15,758 (6,446, 28,389)
Fold-increase IgG^3^	23 (12, 40)

^a^n (%); Median (IQR)

^b^CTV_H = Clinical Target Volume Highest

^c^AZD1222 = AstraZeneca vaccine, BNT162b2 = Pfizer-BioNTech vaccine, mRNA-1273 = Moderna vaccine

^d^8 patients lost to follow-up

**Fig. 1:**
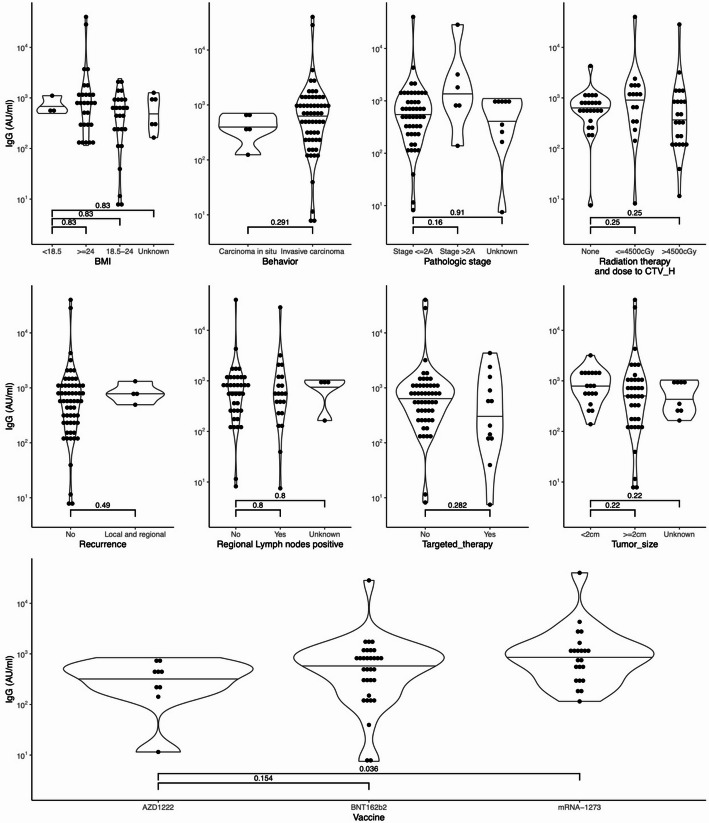
Univariate analysis of Anti-SARS-CoV-2 antibody titers following initial vaccination across various factors. Intergroup comparisons were made using the Wilcoxon rank-sum test, with p-values adjusted for multiple comparisons using the Benjamini-Hochberg method. *IgG* Immunoglobulin G, *BMI* body mass index, *cGy* centigray, *CTV_H* high-risk clinical target volume. The Wilcoxon rank-sum test for intergroup comparisons, and the resulting p-values were adjusted for multiple comparisons using the Benjamini-Hochberg method

**Fig. 2:**
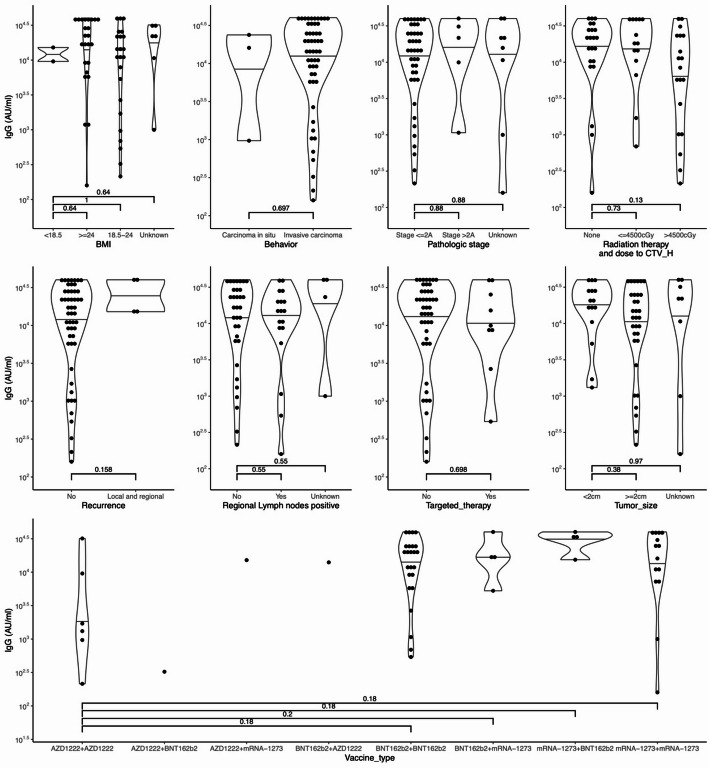
Univariate statistical analysis of Anti-SARS-CoV-2 antibody titers postsecond dose by various nonvaccine factors. Intergroup comparisons were made using the Wilcoxon rank-sum test, with p-values adjusted for multiple comparisons using the Benjamini-Hochberg method. *IgG* Immunoglobulin G, *BMI* body mass index, *cGy* centigray, *CTV_H* high-risk clinical target volume. The Wilcoxon rank-sum test for intergroup comparisons, and the resulting p-values were adjusted for multiple comparisons using the Benjamini-Hochberg method

The median anti-SARS-CoV-2 IgG antibody titers following initial COVID-19 vaccination were 661 AU/mL (IQR: 310-1,419) in the healthy control group and 610 AU/mL (IQR: 258-1,082) in breast cancer patients. Multivariable analysis revealed that breast cancer patients achieved comparable seroconversion rates following COVID-19 vaccination to those observed in healthy controls (Table 2). The median age for the healthy control group is 48 years (IQR: 38–63), and for the breast cancer patient group, it is 51 years (IQR: 46–57). Our analysis indicates that age did not significantly affect the effectiveness of initial COVID-19 vaccination on seroconversion (*p* = 0.060, Table 2), though this marginally significant value suggests a potential trend that warrants further investigation with a larger sample. Various factors influencing seroconversion efficacy during immunization were considered, including body mass index (BMI), whether the carcinoma is in situ or invasive, peripheral lymph node metastasis, the use of chemotherapy, radiation therapy duration and dosage, use of targeted or hormonal therapies, hormone or HER2 receptor status of the tumor, recurrence, and the time elapsed between diagnosis and vaccine administration study. Univariate statistical analysis revealed no statistically significant differences in anti-SARS-CoV-2 antibody titer after the first vaccination among the above factors (Fig. 1). This held for most vaccine brands, highlighting the effectiveness of RNA-based vaccines. Nonetheless, among these brands, the anti-SARS-CoV-2 IgG triggered by mRNA-1273 was notably more effective than that produced by AZD1222 (Fig. 1, *p* = 0.032). However, no significant difference was observed in anti-SARS-CoV-2 IgG levels between the AZD1222 and BNT162b2 vaccines (Fig. 1, *p* = 0.154). These results align with other studies, indicating that lipid-encapsulated mRNA-1273 targeting the COVID-19 spike protein outperformed the delivery method using a replication-deficient adenoviral vector for AZD1222. When evaluating anti-SARS-CoV-2 IgG titer levels post second dose, no notable differences in IgG levels emerged in the univariate analysis, which is anticipated considering the efficacy of the initial vaccine dose (Fig. 2).

**Table 2:** Effectiveness of initial COVID-19 vaccination on seroconversion in breast cancer patients and healthy cohort.^a^

Characteristic	Healthy cohort / breast cancer patient	Beta	95% CI	*p*-value
Vaccine^b^, N (%)				
AZD1222	21 (49%) / 9 (14%)	–	–	
BNT162b2	4 (9.3%) / 32 (51%)	0.935	-2.42, 4.29	0.586
mRNA-1273	17 (40%) / 22 (35%)	1.99	-1.30, 5.28	0.239
Unknown	1 (2.3%) / 0	2.74	-4.62, 10.1	0.467
Age, median (IQR)	48 (38, 63) / 51 (46, 57)	-0.049	-0.100, 0.001	0.060
Patient cohort, N				
Healthy control	43	–	–	
Breast cancer	63	0.883	-0.44, 2.21	0.195

^a^Multivariable linear regression analysis was performed with adjustment for vaccine brands and age. The regression model used log-transformed IgG titers as the dependent variable. Beta coefficients represent the adjusted difference in log-transformed IgG titers between groups

^b^AZD1222 = AstraZeneca vaccine, BNT162b2 = Pfizer-BioNTech vaccine, mRNA-1273 = Moderna vaccine

To assess whether any of the mentioned factors affected seroconversion efficiency post-COVID-19 vaccination, we conducted a multivariate statistical analysis focusing on factors pertinent to breast cancer patient’s prognosis and clinical outcomes. Due to variations in anti-SARS-CoV-2 IgG potency following the initial vaccine dose, all variables, including the vaccine brand, were considered (Table 3). Notably, our findings indicate that chemotherapy use, regional lymph node tumor involvement, and the left lateral location of the tumor adversely affected antibody titer uptake following the first COVID-19 vaccine dose vaccine (Table 3). Factors positively associated with higher antibody titer values following COVID-19 vaccination included tumor size less than 2 cm, stage > 2 A disease, ER receptor presence, and radiation therapy use (Table 3). Some of this evidence may be linked to surgery, as stage 2 disease is less likely to undergo surgical intervention. Additionally, these patients may be younger women with ER-negative disease, with age contributing to better viral uptake. These findings support the idea that ongoing immunizations should only prioritize those with preexisting conditions.

The multivariate analysis of antibody titers following the second dose of the COVID-19 vaccine shows that only patients with a longer time between diagnosis and vaccination had significantly higher sera IgG levels than those with a shorter interval (Table 4). No differences were observed in the anti-SARS-CoV-2 IgG titers when comparing brands or combinations of the first and second doses (Table 4). We conducted a multivariate statistical analysis to examine factors influencing the prognosis and clinical outcomes for breast cancer patients, specifically focusing on the seroconversion efficiency of the second COVID-19 dose. Our study indicated that negative factors impacting the relative fold change in seroconversion after the first dose included radiation or hormonal therapy (Table 5). The positive factors influencing seroconversion included age, tumor size > 2 cm, the presence of the ER receptor, and a prior surgical history (Table 5). Ultimately, we gathered data from the side effects questionnaire completed by breast cancer patients. The most commonly reported side effects were pain at the injection site (28.6%) and fatigue (22.2%) (Table 6).

**Table 3:** Effectiveness of initial COVID-19 vaccination on seroconversion in breast cancer patients

Characteristic	Beta	95% CI^a^	*p*-value
Age	0.025	-0.088, 0.138	0.670
BMI			
< 18.5	–	–	
≧ 18.5 and < 24	-11.0	-24.2, 2.19	0.111
≧ 24	-11.1	-25.4, 3.21	0.138
Unknown	-14.5	-33.0, 4.08	0.136
Laterality			
Right	–	–	
Left	-3.85	-6.86, -0.829	0.017
Behavior			
Carcinoma in situ	–	–	
Invasive carcinoma	-7.77	-21.0, 5.50	0.259
Tumor size			
< 2 cm	–	–	
≧ 2 cm	5.13	4.24, 6.02	< 0.001
Unknown	-2.71	-15.1, 9.69	0.671
Regional lymph nodes positive			
No	–	–	
Yes	-3.95	-6.05, -1.86	0.001
Unknown	-9.60	-18.5, -0.647	0.043
Pathologic stage			
Stage ≦ 2 A	–	–	
Stage > 2 A	3.84	1.58, 6.09	0.002
Unknown	21.6	5.52, 37.6	0.013
Recurrence			
No	–	–	
Local and regional	2.57	-0.110, 5.26	0.069
ER			
Negative	–	–	
Positive	6.71	2.13, 11.3	0.007
Unknown	15.1	-21.6, 51.8	0.426
PR			
Negative	–	–	
Positive	1.19	-11.7, 14.1	0.858
Unknown			
HER2			
Negative	–	–	
Positive	-1.33	-4.51, 1.86	0.420
Unknown	-9.23	-45.2, 26.7	0.618
Surgery			
No	–	–	
Yes	16.6	-0.98, 34.2	0.073
Surgical margins of the primary site			
No	–	–	
Yes	-3.00	-9.11, 3.12	0.344
Radiation therapy and dose to CTV_H^b^			
No radiation therapy	–	–	
≦ 4500 cGy	5.94	2.21, 9.67	0.004
> 4500 cGy	6.82	2.91, 10.7	0.002
Chemotherapy			
No	–	–	
Yes	-9.00	-13.6, -4.37	0.001
Hormone therapy			
No	–	–	
Yes	5.52	-9.07, 20.1	0.464
Targeted therapy			
No	–	–	
Yes	9.15	4.52, 13.8	< 0.001
Time from diagnosis to vaccination (years)	2.15	0.888, 3.40	0.002
Vaccine brands^c^			
AZD1222	–	–	
BNT162b2	5.52	-6.66, 17.7	0.381
mRNA-1273	12.4	-1.38, 26.1	0.087

^a^CI = Confidence Interval

^b^CTV_H = Clinical Target Volume Highest

^c^AZD1222 = AstraZeneca vaccine, BNT162b2 = Pfizer-BioNTech vaccine, mRNA-1273 = Moderna vaccine

**Table 4:** Evaluation of seroconversion rates following the second dose of COVID-19 vaccination in patients with breast cancer

Characteristic	Beta	95% CI^a^	*p*-value
Age	0.068	-0.026, 0.162	0.172
BMI			
< 18.5	–	–	
≧ 18.5 and < 24	-2.20	-7.46, 3.06	0.423
≧ 24	-2.94	-8.39, 2.52	0.303
Unknown	-3.84	-10.7, 3.00	0.284
Laterality			
Right	–	–	
Left	-0.185	-0.99, 0.620	0.657
Behavior			
Carcinoma in situ	–	–	
Invasive carcinoma	0.028	-2.06, 2.12	0.98
Tumor size			
< 2 cm	–	–	
≧ 2 cm	0.083	-0.580, 0.746	0.808
Unknown	3.62	-0.882, 8.12	0.131
Regional lymph nodes positive			
No	–	–	
Yes	-0.859	-1.74, 0.025	0.071
Unknown	-2.08	-5.93, 1.77	0.302
Pathologic stage			
Stage ≦ 2 A	–	–	
Stage > 2 A	-0.737	-2.17, 0.691	0.324
Unknown	0.444	-2.60, 3.49	0.778
Recurrence			
No	–	–	
Local and regional	0.649	-1.27, 2.57	0.515
ER			
Negative	–	–	
Positive	0.650	-1.47, 2.77	0.554
Unknown	1.36	-47.1, 49.8	0.96
PR			
Negative	–	–	
Positive	1.54	-4.87, 7.95	0.643
Unknown			
HER2			
Negative	–	–	
Positive	1.80	-0.284, 3.88	0.106
Unknown	-3.18	-52.1, 45.8	0.900
Surgery			
No	–	–	
Yes	2.04	-3.32, 7.40	0.464
Surgical margins of the primary site			
No	–	–	
Yes	2.78	0.069, 5.48	0.058
Radiation therapy and dose to CTV_H^b^			
No radiation therapy	–	–	
≦ 4500 cGy	0.702	-0.191, 1.59	0.139
> 4500 cGy	0.247	-0.844, 1.34	0.662
Chemotherapy			
No	–	–	
Yes	-3.64	-7.68, 0.408	0.093
Hormone therapy			
No	–	–	
Yes	2.91	-1.25, 7.07	0.185
Targeted therapy			
No	–	–	
Yes	-0.136	-1.87, 1.60	0.879
Time from diagnosis to vaccination (years)	0.436	0.093, 0.779	0.022
Vaccine type^c^			
AZD1222 + AZD1222	–	–	
AZD1222 + BNT162b2	-1.84	-81.6, 77.9	0.96
AZD1222 + mRNA-1273	0.939	-2.91, 4.79	0.638
BNT162b2 + BNT162b2	2.81	-1.51, 7.13	0.217
BNT162b2 + AZD1222	2.60	-2.19, 7.39	0.301
BNT162b2 + mRNA-1273	2.34	-1.98, 6.65	0.301
mRNA-1273 + mRNA-1273	2.51	-1.95, 6.97	0.284
mRNA-1273 + BNT162b2	0.894	-3.98, 5.76	0.723
IgG after the first dose	0.000	0.000, 0.000	0.513

^a^CI = Confidence Interval

^b^CTV_H = Clinical Target Volume Highest

^c^Vaccine combinations indicate mixed regimens for first and second doses. AZD1222 = AstraZeneca vaccine, BNT162b2 = Pfizer-BioNTech vaccine, mRNA-1273 = Moderna vaccine

**Table 5:** Effect of second Anti-SARS-CoV-2 IgG dose on folds in breast cancer patients: assessment comparing postfirst dose titer levels

Characteristic	Beta	95% CI^a^	*p*-value
Age	0.165	0.028, 0.302	0.029
BMI			
< 18.5	–	–	
≧ 18.5 and < 24	-1.82	-8.51, 4.87	0.600
≧ 24	-1.75	-8.60, 5.09	0.621
Unknown	-2.97	-11.6, 5.62	0.506
Laterality			
Right	–	–	
Left	0.142	-0.471, 0.755	0.654
Behavior			
Carcinoma in situ	–	–	
Invasive carcinoma	-0.413	-3.04, 2.21	0.761
Tumor size			
< 2 cm	–	–	
≧ 2 cm	1.71	0.405, 3.02	0.018
Unknown	-0.211	-2.85, 2.43	0.877
Regional lymph nodes positive			
No	–	–	
Yes	0.616	-0.134, 1.37	0.123
Unknown	-0.446	-4.03, 3.14	0.810
Pathologic stage			
Stage ≦ 2 A	–	–	
Stage > 2 A	-2.40	-4.67, -0.129	0.052
Unknown	0.522	-1.35, 2.40	0.592
Recurrence			
No	–	–	
Local and regional	-0.788	-2.18, 0.604	0.280
ER			
Negative	–	–	
Positive	1.43	0.099, 2.77	0.048*
Unknown	-0.323	-3.00, 2.35	0.815
PR			
Negative	–	–	
Positive	0.586	-0.930, 2.10	0.458
Unknown			
HER2			
Negative	–	–	
Positive	2.05	-0.761, 4.86	0.168
Unknown	2.41	-8.22, 13.0	0.662
Surgery			
No	–	–	
Yes	5.77	0.353, 11.2	0.050
Surgical margins of the primary site			
No	–	–	
Yes	-1.53	-9.33, 6.27	0.705
Radiation therapy and dose to CTV_H^b^			
No radiation therapy	–	–	
≦ 4500 cGy	-2.73	-4.38, -1.07	0.004
> 4500 cGy	-1.90	-3.42, -0.374	0.024
Chemotherapy			
No	–	–	
Yes	-2.64	-5.78, 0.497	0.115
Hormone therapy			
No	–	–	
Yes	-1.79	-3.15, -0.430	0.018*
Targeted therapy			
No	–	–	
Yes	-2.65	-5.68, 0.382	0.102
Time from diagnosis to vaccination (years)	0.098	-0.225, 0.421	0.557
Vaccine type^c^			
AZD1222 + AZD1222	–	–	
AZD1222 + BNT162b2	-1.07	-20.2, 18.1	0.914
AZD1222 + mRNA-1273	-1.95	-5.09, 1.20	0.239
BNT162b2 + BNT162b2	2.65	-3.33, 8.62	0.396
BNT162b2 + AZD1222	-0.481	-7.29, 6.32	0.891
BNT162b2 + mRNA-1273	2.79	-3.41, 9.00	0.388
mRNA-1273 + mRNA-1273	-1.53	-7.99, 4.94	0.649
mRNA-1273 + BNT162b2	-2.72	-9.60, 4.15	0.447
IgG after the first dose	0.000	0.000, 0.000	0.99

^a^CI = Confidence Interval

^b^CTV_H = Clinical Target Volume Highest

^c^AZD1222 = AstraZeneca vaccine, BNT162b2 = Pfizer-BioNTech vaccine, mRNA-1273 = Moderna vaccine

**Table 6:** Reported side effects of vaccination in breast cancer patients

	*N* (%)
Pain in the injection site	18 (28.6)
Fatigue	14 (22.2)
Headache	8 (2.7)
Muscle pain	7 (11.1)
Swelling in the injection site	7 (11.1)
Fever	6 (9.5)
Chill	3 (4.8)
Joint pain	2 (3.2)
Severe side effect	1 (1.6)
Vomiting	0 (0)

## Discussion

It has been reported that advanced age (over 65 years) is a recognized risk factor for reduced immunogenicity, particularly with adenoviral vaccines. For example, a study of adenovirus vaccine recipients aged 65 years and over demonstrated poorer antibody responses [14]. The non-significant age effect observed in healthy subjects and those with breast cancer in our study may be due to the strict exclusion criteria (e.g. no prior SARS-CoV-2 infection) and the younger age distribution (median 48–51 years, IQR 38–63, see Table 2). This likely mitigates the effect compared to studies involving older populations. Similar age distributions and potential underpowering due to sample size (*n* = 63) are noted as limitations, suggesting that future studies with larger cohorts could clarify age-related trends and elucidate the factors driving our findings further.

Our study provides novel insights into stage-specific vaccine responses in breast cancer, identifying higher seroconversion in Stage > 2 A patients and the critical role of diagnosis-to-vaccination timing, findings with implications for personalized immunization and immunotherapy strategies. This investigation was based on an analysis of 63 participants. Unlike the broader cancer population, where seroconversion rates are significantly lower than in healthy individuals (60% vs. 95% after two mRNA doses [15]), our breast cancer cohort demonstrated robust antibody responses. Results of this study demonstrate that breast cancer patients can mount meaningful antibody responses to the vaccines developed to combat the novel coronavirus. This finding is supported by a large-scale Malaysian study [16] that reported cancer patients achieved high seroconversion rates of 95.1% after the second dose of vaccination, with maintained antibody responses (mean log IgG: 7.07 for BNT162b2 vaccine) at 6 months. However, that study also noted that antibody titers in cancer patients were significantly lower than those in healthy controls at six months after vaccination. These findings, derived from diverse Asian populations, highlight that while vaccination is effective in cancer patients, ongoing monitoring of vaccine responses may be warranted.

A salient finding was the marked superiority of mRNA vaccines, particularly mRNA-1273, in eliciting robust antibody responses when compared to the adenoviral vector vaccine (AZD1222). This finding is consistent with the findings of recent studies [17] and meta-analyses [18–20] that have demonstrated that mRNA vaccines tend to elicit more robust immune responses compared to viral vector vaccines. A subsequent meta-analysis [21] further corroborated the notion that mRNA vaccines elicited more robust immune responses across a range of vaccine types. However, it is noteworthy that cancer patients, including those with breast cancer, exhibit diminished immune responses to vaccines in comparison to healthy individuals [22].

However, the unexpected higher seroconversion in patients with advanced disease stages (≥ 2 A) after the first vaccine dose (Table 3) and the absence of stage-related differences post-second dose (Table 4) warrant further exploration, as do the broader immunological dynamics in breast cancer. The higher IgG titers in Stage > 2 A patients after the first vaccine dose (Table 3) are particularly intriguing, given that advanced stages are typically associated with intensive treatments like chemotherapy, which suppressed titers in our cohort (Table 3). This paradox may be explained by enhanced Th2 responses. IL-4, which is correlated with poor outcome of breast cancer [23], promotes B-cell activation and antibody production, potentially driving stronger initial seroconversion in Stage > 2 A patients. Additionally, immune recovery in patients with longer diagnosis-to-vaccination intervals (Table 4) or prior radiation therapy (Table 3) may outweigh chemotherapy’s suppressive effects. For instance, radiation was positively associated with titers (Beta = 5.94–6.82, *p* ≤ 0.004), possibly due to the driven of Th2 polarization of CD4 + T cells by radiation treatment [24]. However, the small Stage > 2 A sample size (*n* = 6) and lack of direct IL-4 measurements limit these conclusions, and unmeasured confounders, such as younger age, may contribute. The absence of stage-related differences after the second dose (Table 4) likely reflects a ceiling effect, where additional vaccination maximizes antibody responses across all stages, or cumulative treatment-related suppression neutralizing Th2 advantages in advanced stages.

The association of ER positivity with higher IgG titers (Table 3) may reflect differences in treatment intensity or tumor biology. ER-positive breast cancers are often treated with hormonal therapies, which are less immunosuppressive than chemotherapy, potentially preserving immune responses [9, 25]. In contrast, ER-negative patients, particularly those with triple-negative disease, may receive more dose-intense chemotherapy regimens, which could suppress antibody production, as chemotherapy was negatively associated with titers (Table 3). The lack of data on chemotherapy dose intensity in our study limits our ability to confirm this confounding effect, but it aligns with clinical patterns where ER-negative disease requires aggressive treatment [25]. Future studies should explore chemotherapy regimens and ER status to clarify their impact on vaccine responses.

Regarding the safety and immunogenicity of COVID-19 vaccines in breast cancer patients on active anticancer therapy, recent studies provide supportive evidence. Zhang et al. reported similar mild adverse event rates to the general population, regardless of vaccine type or timing, with enhanced safety in early-stage patients [26]. Fernández-Murga et al. demonstrated robust antibody and memory T-cell responses to mRNA vaccines, though over half lacked antigen-specific CD8 + T cells, indicating complementary humoral and cellular immunity [27]. A meta-analysis by Thöne et al. confirmed high seroconversion rates, particularly with radiotherapy alone, but noted reduced immunogenicity during chemotherapy or immunosuppression, recommending booster doses and monitoring [28]. These findings underscore the vaccines’ overall tolerability while highlighting the need for tailored strategies in immunosuppressed subgroups.

Lymph node involvement and ongoing chemotherapy were identified as negative factors that could diminish vaccine effectiveness. Treatment timing and type were found to have a significant impact, with prior treatments, especially chemotherapy and hormone therapy, adversely affecting antibody levels. This suggests that ongoing cancer therapies may compromise immune system function and reduce vaccination effectiveness, aligning with findings from Tantiyavarong et al. [29], who observed significantly lower antibody titers in chemotherapy patients post-vaccination. These findings complement the work of Zhang et al. [30], who investigated post-Omicron breakthrough infection responses with inactivated vaccines, though their study did not explore these specific clinicopathological factors. With regard to safety, the observed side effects were generally mild, primarily consisting of injection site pain and fatigue, consistent with typical vaccine responses in the general population, as highlighted in a review by Juhel et al. [16].

Based on these comprehensive findings, we put forward several recommendations to enhance vaccination strategies for breast cancer patients. First, the optimal timing for vaccination merits careful consideration, with a potential recommendation of postponing immunization until after a patient’s initial diagnosis and treatment to ensure maximal effectiveness. Secondly, the selection of vaccine type emerges as a pivotal consideration, with mRNA vaccines demonstrating superior immunogenicity compared to adenoviral vector vaccines in this demographic. Furthermore, individual clinical profiles, including tumor characteristics and treatment regimens, should inform vaccination approaches.

The integration of our findings with previous research, particularly the complementary evidence from Zhang et al.‘s study [30], underscores the complexity of immune responses in breast cancer patients and the necessity for personalized vaccination strategies. While the present study focused on immediate post-vaccination responses with mRNA and viral vector vaccines, the combined evidence suggests that both vaccine type and individual clinicopathological profiles play crucial roles in determining vaccination outcomes. This comprehensive understanding may help optimize vaccination strategies for this vulnerable population, particularly regarding the timing of vaccination in relation to diagnosis and treatment schedules.

### Limitations

This study has several limitations that should be acknowledged. First, reliance on cancer registry data restricted our ability to stratify analyses by detailed molecular subtypes (e.g., Luminal A, Luminal B, HER2-enriched, Basal-like/TNBC) or specific chemotherapy regimens (e.g., anthracycline- or taxane-based), as only binary indicators (e.g., ER/PR/HER2 status and chemotherapy receipt) were available. The lack of tissue samples from the registry also precluded analysis of proliferation markers like Ki-67, limiting subtype stratification. Additionally, the absence of detailed chemotherapy status (e.g., specific regimens or timing relative to vaccination) limited our ability to confirm findings from prior studies, such as lower seropositivity and antibody titers in cancer patients on active chemotherapy reported by Ligumsky et al. for BNT162b2 [31] and reduced anti-S IgG titers in patients with solid tumors undergoing chemotherapy after ChAdOx1 nCoV-19 or CoronaVac vaccination noted by Tantiyavarong et al. [29]. This may limit the generalizability of our findings to subtype-specific immune responses. Second, while we accounted for mixed vaccine regimens due to availability during the enrollment period, the sample size for certain combinations was small, potentially underpowering subgroup analyses despite no significant differences observed. Third, the vaccine type distribution differed between healthy controls and breast cancer patients, with a higher proportion of healthy controls receiving AZD1222 and the majority of cancer patients receiving mRNA vaccines (BNT162b2 or mRNA-1273). Although multivariate regression adjusted for this disparity, the uneven distribution may introduce residual confounding, potentially influencing the observed similarities in antibody titers, as noted in Table 2. Fourth, the heterogeneity in vaccine schedules and limited IgG subtype data further constrain long-term immunity assessment. Fifth, antibody titers were assessed at fixed post-vaccination intervals (third week after first dose and second week after second dose), which may not capture long-term dynamics or booster effects. Additionally, self-reported side effects via questionnaires could introduce recall bias. Finally, the study was conducted in a single-center setting in Taiwan, which may not fully represent diverse populations or evolving SARS-CoV-2 variants. Future studies with larger, multi-center cohorts and more granular data (e.g., from electronic health records) could address these gaps and further elucidate factors influencing vaccine immunogenicity in breast cancer patients.

## Conclusion

This study highlights the varying immune responses to the Pfizer-BioNTech BNT162b2 vaccine in breast cancer patients. Stronger antibody levels were observed after the first dose in patients with advanced disease (stage > 2 A), larger tumors (≥ 2 cm), ER-positive status, or prior radiation therapy. However, these differences diminished after the second dose. These results imply the potential advantages of personalized vaccination strategies adapted to disease stage and treatment timing, particularly given that chemotherapy was found to suppress antibody levels, while radiation therapy was positively associated with them. However, the conclusions remain observational due to several limitations. These include the small sample size (*n* = 63), reliance on cancer registry data that lacks subtype or proliferation markers (e.g. Ki-67) and heterogeneity in vaccine schedules, which may not reflect long-term immunity. The lack of experimental data, such as on IgG subclasses or cytokine profiles, further restricts the ability to draw causal inferences about mechanisms such as the hypothesized Th2 bias. Furthermore, the association with radiation is based on a limited subgroup analysis. These constraints underscore the need for caution when generalizing the results. Future research should explore larger cohorts with detailed stratification—including precise documentation of chemotherapy regimens such as anthracycline-based, taxane-based, and cyclophosphamide-containing therapies—which could facilitate case-based analyses of specific chemotherapy types associated with impaired vaccine responses. Incorporation of experimental assays will help to validate immune dynamics and vaccine memory. Meanwhile, policymakers might consider integrating these insights into vaccination guidelines, potentially recommending optimized timing post-treatment. However, further evidence is needed to confirm the effects of different vaccine types or schedules. This approach could enhance protection for breast cancer patients while addressing evolving public health challenges. These insights could serve as a paradigm for optimizing vaccine administration in breast cancer patients against other infectious diseases, leveraging disease stage and treatment timing.

## Data Availability

Data are available from the Research Ethic Committee of Chung Shan Medical University Hospital for researchers who meet the criteria for access to confidential data. Requests for the data may be sent to the Research Ethic Committee, Chung Shan Medical University Hospital.

## Abbreviations

BMI: Body mass index
CDK: Cyclin-dependent kinase
COVID-19: Coronavirus disease 2019
ER: estrogen receptor
HER2: Human epidermal growth factor receptor 2
IQR: Interquartile range
PCR: Polymerase chain reaction
PR: Progesterone receptor
SARS-Cov-2: Severe acute respiratory syndrome Coronavirus 2
TNBC: Triple-negative breast cancer
